# Exclusive breastfeeding and HIV/AIDS: a crossectional survey of mothers attending prevention of mother-to-child transmission of HIV clinics in southwestern Nigeria

**DOI:** 10.11604/pamj.2015.21.309.6498

**Published:** 2015-08-27

**Authors:** Usman Aishat, Dairo David, Fawole Olufunmilayo

**Affiliations:** 1 Nigeria Field Epidemiology and Laboratory Training Programme, Abuja, Nigeria; 2 Department of Community Medicine, LAUTECH Teaching Hospital, Osogbo, Osun State, Nigeria; 3 Department of Epidemiology and Medical statistics, University of Ibadan, Oyo State, Nigeria

**Keywords:** Mother-to-child transmission, exclusive breastfeeding, HIV clinics

## Abstract

**Introduction:**

Prevention of Mother-To-Child-Transmission (PMTCT) of Human Immunodeficiency Virus (HIV) guideline recommends replacement feeding where it is acceptable, feasible, affordable, sustainable and safe. Where this is un-achievable, exclusive breastfeeding (EBF) is recommended during the first six months of life.

**Methods:**

A hospital-based cross-sectional study was conducted among 600 HIV-positive using a two-stage sampling technique. Data on socio-demographics, infant feeding choice and factors influencing these choices were collected using semi-structured questionnaires.

**Results:**

Majority of the mothers (86.0%) were married and aged 31.0 ± 5.7years. Slightly above half (53.0%) had≤2 children and more than two-third had disclosed their HIV status to their spouses. About two-third (61.0%) were traders with 75.0% earning monthly income ≤N5,000.00k. Half of the mothers had ≥4 antenatal care visits and 85.0% had infant feeding counselling. Infant feeding choices among the mothers were EBF (61.0%), ERF (26.0%) and MF (13.0%). The choice of EBF was influenced by spouse influence (84.0%), family influence (81.0%) and fear of stigmatisation (53.0%). Predictors of EBF were; monthly income (AOR = 2.6, C.I. =1.4-4.5), infant feeding counselling (AOR = 2.7, C.I. = 1.6-6.9) and fear of stigmatisation (AOR = 7. 2, C.I. = 2.1-23.6).

**Conclusion:**

HIV positive mothers are faced with multiple challenges as they strive to practice exclusive breastfeeding. More extensive and comprehensive approach of infant feeding counseling with emphasis on behavioural change programmes in the context of HIV/AIDS within communities is advocated.

## Introduction

Breastfeeding plays a major role in nutrition, health and development for both HIV infected and non HIV infected infants, due to the fact that human milk is the ideal nourishment for infants' survival, growth and development [1]. When the infants are exclusively breastfed for the first six months of life, there is stimulation of immune system and this goes hand in hand with protecting them from diseases like diarrhoea and acute respiratory infections, which are two of the major causes of infant mortality in the developing world [1]. When exclusive breast-feeding is practice, there is a lower risk of HIV transmission than mixed feeding [2, 3]. Exclusive Breast Feeding (EBF) is giving the infant no other food or drink, not even water, apart from breast milk (including expressed breast milk), with exception of drops or syrups consisting of vitamins, mineral supplements or prescribed medicine; if it is practice for the first six months of an infant′s life, is a beneficial intervention in saving children′s lives [4, 5]. Despite the benefits which results from its practice, EBF rates remain low throughout the world, where globally the rate of exclusive breastfeeding is around 35% [6]. Different regions in the world have reported increase of EBF, for instance from 22% (1996) to 30% (2006) in sub-Saharan Africa, East Asia /Pacific, (excluding China) 27% (1996) to 32% (2006) and in Latin America and the Caribbean, (excluding Brazil, and Mexico) 30% to 45%, despite the reported increase of EBF, the rates are still low [4]. However; according to Nigeria Demographic and Health Survey 2013, only 17.0% of infants at the age below 6 months regardless of their HIV status are exclusively breastfed [6]. This rate suggests some improvement from the previous Nigeria Demographic and Health Survey of 2008 which indicated that only 13% were exclusively breastfed, still the rates are low with observed variations. Transmission of HIV from mother to child is higher among the mixed fed infants than exclusively breast-fed infants. It is estimated that with Exclusive Breastfeeding (EBF) practice, 13% to 15% deaths of children below 5 years of age could be averted in low and middle-income countries [7]. WHO recommends EBF to both HIV exposed and non exposed infants for the first six months of life, but still EBF rates remain low throughout the world. Globally, the estimated prevalence of exclusive breastfeeding is 35% [8]. Thus, this study intends to add the knowledge on the observed gap in this area by assessing factors influencing EBF practice among HIV positive mothers in Oyo State. The findings of this study are expected to tell policy decision makers to develop right interventions to promote exclusive breastfeeding hence improvement of child health in Oyo State and Nigeria.

## Methods

**Study area**: We conducted a cross-sectional study in PMTCT clinics in Oyo State between 2012 and 2013. There are three senatorial zones in Oyo State namely Oyo Central, Oyo South and Oyo North with 10 PMTCT clinics. The location of the PMTCT clinics within the zones is as follows; Oyo Central-2, Oyo South-5, Oyo north-3. **Study population** : The respondents who were eligible to take part in the study included; HIV positive mothers who participated in the PMTCT programme in the 6 facilities, mothers who had babies between the age of 6 weeks to 12 months and mothers attending PMTCT clinics for a period not less than six months before the study. We excluded mothers or infants who were too sick from the study. **Sample size determination**: We calculated the sample size using formula for single proportion [9] with prevalence of 0.68 [10] and 10% non-response rate, the sample size was 600. **Sampling procedure**: A two-stage sampling technique was used. In stage 1: The list of all the PMTCT clinics in each senatorial zone was obtained, and stratified into three senatorial zones. We used simple random sampling to select 6 clinics out of the 10 in the State using proportional allocation. We selected three clinics from Oyo South senatorial zone,1 clinic from Oyo Central zone and 2 clinics from Oyo North senatorial zone. Stage 2: The last 6 months' patient load list from each of the PMTCT clinics was gotten and the total client load in the 6 facilities was the sampling frame. Respondents were proportionately allocated based on patients load. The first mother in each clinic was randomly selected afterwards; every 4th mother selected using systematic sampling until we arrived at desired sample size per facility. **Study variables** :The dependent variable was mother's infant feeding practice (Exclusive Breast Feeding, Exclusive Replacement Feeding, and Mixed Feeding) while the independent variables were age, marital status, education and occupation status of mothers, monthly income, disclosure of HIV status, place of delivery, mode of delivery, antenatal care visits, gestational age at first antenatal care visit. **Data collection**: Four trained research assistants collected data from the selected PMTCT clinics for a period of four weeks using interviewer administered semi-structured questionnaire. Questionnaire consisted of 4 sections namely: Socio-demographics, knowledge of mothers on exclusive breastfeeding, infant feeding practices and factors influencing choice of exclusive breastfeeding practice. **Data analysis**: We computed descriptive statistics using Epi-info Version 7.0 (CDC, 2007) to generate summary statistics. Bivariate analyses were done to measure association between independent variables and infant feeding options using crude odd's ratio. The p < 0.05 was used to find level of statistical significance. Logistic regression model was fitted to find factors influencing choice of infant feeding practice by HIV positive mothers. **Ethical consideration**: Ethical Review Committee of Oyo State Ministry of Health gave ethical clearance for this study. Mothers gave written informed consent. In order to make sure there is confidentiality of any information provided, the data collection procedure was anonymous.

## Results

**Figure 1:**
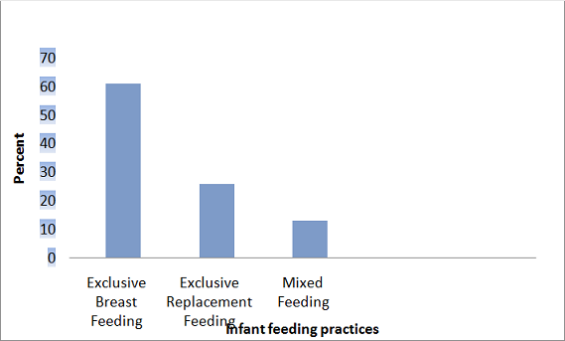
Infant feeding practices of mothers

**Background characteristics of HIV positive mothers**: Table 1 Shows background characteristics of HIV positive mothers. The mean age of the mothers was 31.0 ± 5.7years. Majority of the mothers (86.0%) married and (80.0%) had babies less than six months of age. More than half had (53.0%) had two children or less. Two hundred and fifty-three mothers (42.0%) completed secondary school education and their main occupation was trading. Three hundred and forty-three (57.0%) were Christians. **Knowledge of HIV mothers on exclusive breast-feeding**: Table 2 Shows two categories of mothers, first group: that is having good knowledge on EBF if a mother could define EBF properly (that is how to do it correctly and its duration), second group: having poor knowledge if she could not define it properly. In this regard, mothers who were able to define it as breastfeeding only without any other food or liquid for first six months of infant's life had good knowledge on EBF. The results show that most 158(79%) knew what EBF is, with few (16.2%) though managed to defined how it is done correctly, missed and reported the duration for EBF as four and three months, where (4.8%) reported not knowing what it is as indicated in Table 2. **Infant feeding Practices of HIV positive mothers**: Figure 1 Shows the infant feeding practices of HIV positive mothers. From 600 HIV positive mothers, 480 (80.0%) HIV positive mothers had children with age less than 7 months. Of the 480(80.0%) HIV positive mothers, 293(61.0%) exclusively breastfed their children, 62(12.9%) practiced mixed feeding and 125(26.0%) practiced exclusive replacement feeding. **Reasons for choosing exclusive breast feeding by mothers**: Table 3 Shows the reasons for choosing Exclusive Breast Feeding among the mothers. One hundred and fifty-six (53.0%) chose EBF because they fear stigmatization, one hundred and seventy-six (60.0%) because breastfeeding prevents childhood infections and one hundred and forty-seven because of health worker/counselor's influence. Two hundred and forty-six (84.0%) chose EBF because of their spouse influence, while two hundred and thirty-six (81.0%) was because of family influence. **Factors influencing the choice of exclusive breastfeeding practice** : Table 4 Shows factors influencing the choice of exclusive breastfeeding practice. Mothers who earned less than N18,000 were five times more likely to practice EBF (OR = 4.6, 95% CI = 2.45-41.19). Mothers who had ANC visits more than three times were five times more likely to practice EBF (OR = 4.6, 95% CI = 1.66-32.2). Mothers who had infant feeding counseling were five times more likely to practice EBF (OR = 5.2, 95% CI = 2.69-61.94). Fear of stigmatization was one of the predictors of exclusive breastfeeding. Mothers who fear stigmatization were five times more likely to practice EBF (OR = 5.2, 95% CI = 2.15-13.00).

**Table 1:** Socio-demographic characteristics of respondents

Variables	Frequency (n = 600)	Percent (%)
**Mothers age (years)**		
20- 24	80	13.3
25-29	159	26.5
30-34	200	33.4
≥ 35	161	26.8
**Age of children(months)**		
≤ 6	480	80.0
7-11	84	14.0
≥12	36	6.0
Parity		
≤ 2	318	53.1
3-5	256	42.7
≥ 6	26	4.2
**Educational status**		
None	55	9.2
Primary completed	139	23.1
Secondary completed	253	42.2
Post secondary	153	25.5
**Marital status**		
Married	518	86.3
Single parent/Divorced	46	7.7
Never married	36	6.0
**Occupation**		
Trader/Artisan	364	60.7
Civil servant	135	22.5
Housewife/unemployed	63	10.5
Farmer	38	6.3
**Monthly income (in Naira)**		
< 5,000	498	92.7
5,000-18,000	30	5.6
> 18,000	9	1.7

**Table 2:** Knowledge of study participants on EBF (n = 600)

Variables	Frequency	Percent
Breastfeeding infant without giving any food or liquid for the first six months	474	79.0
Breastfeeding infant without giving any food or liquid for the first four months	66	11.0
Breastfeeding infant without giving any food or liquid for the first three months	31	5.2
I don't Know	29	4.8

**Table 3:** Respondents reasons for choosing exclusive breast feeding

Variables	Frequency (n =293)	Percent
Fear of stigmatization	156	53.3
Breast feeding prevents childhood infections	176	60.1
Health workers/Counselors influence	147	50.2
Spouses influence	246	84.0
Family influence	237	81.0

Note: Multiple responses allowed

**Table 4:** Factors influencing the choice of exclusive breastfeeding practice by mothers

Variables	EBF (293)	COR(95%CI)	AOR(95%CI)	P value
**Income (N)**				0.02^+^
≤ 18,000	276(94.3)	4.6(2.45-41.19)	2.6(1.45-4.59)^+^	
> 18,000	7 (5.7)	ref	ref	
**ANC attendance**				< 0.00^+^
≤ 3times	48(16.4)	ref	ref	
> 3times	245(83.6)	4.6(1.66-32.2)	4.2(1.21-14.9)^+^	
**Infant feeding counseling**				0.01^+^
Yes	234(80.0)	5.2(2.69-61.94)	2.7(1.62 -6.94)^+^	
No	50(20.0)	ref	ref	
**Fear of stigmatization**				0.00^+^
Yes	185(63.1)	5.2(2.15-13.00)	7.2(2.11-23.60)^+^	
No	108(36.9)	ref	ref	
**EBF prevents childhood in infections**				0.30
Yes	176(60.1)	1.41(0.79-2.52)	0.4(0.18-6.84)	
No	117(39.9)	ref	ref	
**Health worker's /c counselor's influence**				0.93
Yes	147(50.2)	ref 2.2(0.82-7.34)	1.7(0.09-3.31)	
No	146(48.2)	ref	ref	

## Discussion

Majority of the mothers (93.0%) had monthly income less or equal N18,000.00k.This similar to finding from Abuja Nigeria where 82.0% of mothers were earning less than N30,000.00k per month [11]. Muko et al. reported that 52.0% of mothers in Cameroon were earning less than $1 per day [12]. This finding suggests that majority of the mothers were of low socio-economic status and this characteristics of most African countries. One of the factors that influenced the choice of exclusive breastfeeding was mother's income. Income influences purchasing power at household level. It affects affordability and access to infants feed [13]. Majority of the mothers interviewed had low monthly income of < N18,000k. Variations in level of income exposed mothers to different levels of purchasing power. There was a statistical significant association between monthly income and practicing EBF. This similar to finding from Kenya by Wapang'ana (2013) where 68.7% of mothers had low annual income of less than Ksh 12,000 and practiced EBF [14]. Another factor that influenced the choice of EBF was the receipt of counseling on infant feeding options during ANC visits. Mothers who received counselling on infant feeding options recommended for HIV positive mothers chose exclusive breastfeeding as an option to feeding their children. This finding is in line with report of Ndubuka et al. where receiving infant feeding counseling was significantly associated with decision to exclusively breast feed. This shows that the counseling had good impact on the mother's choice of infant feeding [13]. Comprehensive and explanatory counseling has the potential to greatly influence mothers' understanding and dedication to exclusive breastfeed and should form the holistic interventions to improve breastfeeding and exclusive breastfeeding rates. Strengthening the counseling being provided during antenatal visits of mothers in health institutions in the study areas and reinforcing counseling of the HIV positive mothers delivered in the maternity wards on safer infant feeding options is recommended as part of the PMTCT program in Oyo State. Number of ANC attendance was also one of the factors that influenced the choice of exclusive breastfeeding. Mothers who attended ANC clinic three or more times were four times likely to practice exclusive breastfeeding that those mothers who did not attend. This also in line with what Hailu (2005) found in Ethiopia [15]. He reported that there was statistical significance relationship between ANC attendance and exclusive breastfeeding. Also Mengistie (2013) reported that mothers who had ANC follow-up were five times more likely to practice exclusive breastfeeding [16]. One possible explanation for this finding is the repeated counseling sessions received by mothers with emphasis on exclusive breastfeeding in the various health facilities. There was association between practicing exclusive breastfeeding and fear of stigmatization. Mothers who fear stigmatization were seven times more seven times more likely to practice exclusive breastfeeding. This finding is similar to findings of Muhammed et al (2010) and Aswa (2010) where mothers practice exclusive breastfeeding were doing so to prevent stigmatization and there was statistical significance between the two [11, 17]. Stigmatization within the community makes HIV mothers prone to the practice of mixed feeding which increases childhood morbidity and mortality.

## Conclusion

This study highlights the factors that contributed to adherence to EBF among HIV positive mothers as: mother's belief that breast milk is enough for infant for the first six months of life, health workers influence on breastfeeding specifically EBF, mother's own decision on infant feeding and health workers facilitate immediate initiation of breastfeeding. Other factors were having knowledge on EBF and believing that if one practices it properly, the MTCT through breast milk is almost non- existing. However, there were barriers like lack of disclosure of one's HIV status, community and family pressure to mix feed as it is a norm, breast problems and contradicting messages of health workers on infant feeding. These findings suggest a need for a more extensive and comprehensive approach of breastfeeding education and especially of exclusive breastfeeding. These important issues related to infant feeding in the context of HIV/AIDS brought up by this study, should taken into account by implementers and policy makers for accelerating exclusive breastfeeding practice among HIV positive mothers. However, since health workers are the sole supporters of infant feeding practices, in particular exclusive breastfeeding, we need to build their capacity to make sure they have current information and positive attitude towards EBF.
